# Hospital and Institutional News

**Published:** 1916-12-30

**Authors:** 


### HOSPITAL AND INSTITUTIONAL NEWS.

### WORK FOR CONVALESCENT SOLDIERS.

AN anonymous writer, who signs himself "Physician at a Military Hospital," has done good service by a letter to the editor of the *Times*, pointing out the immense importance from at least three separate aspects of providing convalescent soldiers with some form of occupation suitable to their strength and capacities. In the first place, as he truly says, such light and graduated exercise as has been abundantly shown to be so valuable in the recovery from pulmonary tuberculosis is equally useful in promoting recovery from other diseases and from wounds; indeed, one may say, especially from wounds, because of the importance of exercising and loosening the stiffened muscles and the partly immobilised joints so frequent as aftermaths of septic gunshot wounds. In the second place, work means relative immunity from those temptations to drink and other things which frequently beset the no longer disabled but not yet entirely recovered soldier, who in many cases falls a prey to them more out of sheer boredom and loneliness than out of any inherent viciousness. Lastly, a supply of labour, even though only part-time and none too strenuous in character, from this source would help towards that economy of the national resources which is at last being recognised by the Government as imperative. So from all these points of view the "Physician's" reminder is timely, and it is to be hoped Mr. Neville Chamberlain and Lord Devonport may bear it in mind when they have time to spare from more important considerations.

### DEATH OF THE FOUNDER OF THE HOSPITAL FOR HIP DISEASE, SEVENOAKS.

ON December 19 the death took place at the Children's Hospital for the Treatment of Hip Disease, Sevenoaks, of the devoted lady who founded that institution and gave to it both of her fortune and of her personal service for forty-four years. Miss Emily Jackson, who was seventy-six years of age, was the daughter of a doctor who devoted the closing years of his life to social and religious work at Sevenoaks, and the sister of Sir Thomas Jackson, R.A. From the exceedingly sympathetic notice of her life in the *Times*, it appears that her first patient was a little girl who could not be nursed properly at home on account of a multitude of brothers and sisters. Miss Jackson's father took a room in a cottage opposite his house, and there his daughter cared for the child. But the little sufferer was lonely, so the next step was to get a second case, from the Great Ormond Street Hospital. Then Miss Jackson, to fit herself better for the work, went to hospital for six months; after which she felt capable of managing four cases. Thus the work grew, and presently, in 1872, the Hospital for Hip Disease came into existence. Rebuilt in 1902 from plans by Sir Thomas Jackson, it now contains thirty-eight beds, and has a budget in the neighbourhood of £1,700 a year. From 1872 until her death "Miss Jackson has given her money, her strength, and her love to the little patients," says the *Times*. She is described as of fine physique, and as constantly regardless of self in her devotion to her life's work. Miss Jackson did not court the limelight, and received no public recognition of her noble services. Probably she desired none; but it is impossible to avoid the feeling that something is very wrong in a community where such devotion can go unrecognised, save by the gratitude of those on whom the benefits are conferred, while honours are showered upon any man who chooses to pay for them. Admittedly, there are few distinctions which the State confers upon women. But the Order of St. John, for instance, would have honoured itself by appointing Miss Jackson to be a Lady of Grace instead of one or other of the numerous society dames who have received that Order during the last few years.

### A NEW CANADIAN CONVALESCENT WAR HOSPITAL.

THE Canadian Red Cross Society has leased and is fitting up St. Lawrence College, at Ramsgate, as a convalescent hospital, with accommodation for one thousand beds. It will bear the name of Princess Patricia, and the Canadian Medical Services will furnish the staff and operate it. A new departure in connection with the latest reforms is that all such hospitals will be operated by the Medical Services. The Red Cross will continue to be responsible for the buildings, the general fabric, and the supply of motor ambulances, but the feeding of the patients will be undertaken by the medical authorities. This is not intended as a reflection upon the Red Cross, but only as an economic reform which will at the same time leave the Red Cross free to develop its work as regards the provision of comforts. The Canadian Convalescent Hospital at Woodcote Park, Epsom, has a new commanding officer, in the person of Captain Alfred Haywood, for two years medical officer of the 3rd Toronto Battalion. The hospital has accommodation for 2,500 patients. Previous to the war Captain Haywood was assistant superintendent of Toronto General Hospital.

### MORE CANADIAN HOSPITAL DEVELOPMENTS.

THE important position of head of the surgical section of the Ontario Military Hospital at Orpington has been accepted by Dr. Haley Williams, of London, Ontario. He is one of Canada's foremost surgeons and is senior clinician of the Western University Medical Department. The appointment comes within the rank of lieut.-colonel. Arrangements have been practically completed between the Military Hospitals Commission and the Ontario Government for the handing over by the Government of the Hospital for the Insane at Whitby (Ontario) to the Commission. It is expected that when the buildings are completed the institution will be the central point for soldiers suffering from shock, and that the institution will be able to take care of 1,200 men. All the men of Military Division No. 2 will be located there.

### MEDICAL MEN AS MINISTERS.

Dr. Christopher Addison, the new Minister of Munitions, is not the only member of the Government who is a qualified medical man. As the *Medical Press* points out, the Lord Chancellor, Sir Robert Finlay, graduated in medicine at Edinburgh University about fifty years ago, but it must have been for only a short period that he practised, for he was called to the English Bar in 1867, and within eighteen years was Solicitor-General, after sitting in Parliament from 1885. At the time of this promotion there was a wicked suggestion going the rounds that preferment was made by the then Prime Minister, Mr. Balfour, less on account of Sir Robert's legal distinction than because of the excellent game of golf that he played.

### GERMAN SUBMARINE BENEFITS HOSPITAL.

It will be remembered that at the time of the exhibition of the German submarine U.C5 at Chatham and Temple Pier, it was announced that the amount raised in admission fees would be divided amongst naval and mercantile marine charities. A report on the subject was last week made in Parliament; the vessel was visited by 320,041 persons, and the total receipts, after expenses had been paid, were £3,639; to this sum was added, for convenience of distribution, £504 collected in Colombo, in the Government Servants' One Day's Pay Fund, for naval charities; this brings the total to be divided up to £4,143. In recognition of the services of the police during the exhibition, the odd £143 was handed to the Metropolitan and City Police Orphanage and £2,000 was given to the Board of Trade, for distribution amongst mercantile marine charities. Various naval orphanages and schools received sums varying from £25 to £400, and one voluntary hospital, St. Bartholomew's, was awarded £50. We are very glad to see our great City hospital benefit by the exhibition, though the precise reason for the grant is not revealed. Perhaps St. Bartholomew's is the nearest hospital to Temple Pier and rendered service in respect of any accidents that may have occurred, or possibly the fact that sailors have been treated at the hospital was taken into consideration. We hope the Board of Trade will not forget the Dreadnought Hospital at Greenwich when the £2,000 in their hands is allocated.

### A PHYSICIAN'S LEGACY TO HIS HOSPITAL.

By the will of the late Dr. Kenneth H. A. Kellie, who was killed in France last summer while serving with the Royal Army Medical Corps, the Belgrave Hospital for Children will receive a legacy of £1,000. Considering that the total estate left by Dr. Kellie amounts to between seven and eight thousand pounds, this legacy deserves the adjective "munificent." It may be taken both as an expression of the testator's affection for this hospital, of which he was one of the visiting medical staff, and as an appreciation from one in the know of the quality of the work done there. Dr. Kellie was educated at Cambridge University and St. George's Hospital, and the Belgrave Hospital appears to have been the sole institution to which he was attached at the time of his acceptance of a commission in the Royal Army Medical Corps.

### A RECORD OF ACTIVITY.

The National Council for Combating Venereal Diseases has issued an interim report dealing with its many activities during the last few months. Some of its suggestions to the Local Government Board, to approved societies, and to other bodies have already been carried into effect; and others are under consideration which, it may be trusted, will be sympathetic. Two of its recommendations have recently been adopted by several local authorities as resolutions to be sent to Whitehall: these are the suppression of advertisements by quacks, and the rendering of the transmission of venereal disease by any person who is aware that he is in an infectious condition a criminal offence. Much educational and propaganda work is being carried on; and it is noteworthy that the Army authorities have been so favourably impressed with the Council's lecture work to soldier audiences that a special request has been made for lecturers to address each class of cadets in the officer-training schools. Institutionally speaking, the most interesting feature of the work of the Council has been the action taken in regard to maternity hospitals. It was discovered by inquiry that the best known of these charities do not willingly receive patients who happen to be suffering from venereal disease. There is nothing wrong in this, since the protection of the other patients is what the hospital authorities have in mind. The rule operates, however, hardly upon these unfortunate women, some of whom have contracted their disease quite innocently, and results in their admission to Poor-Law infirmaries if they need institutional treatment in their confinements. A conference was therefore called, to which representatives of (London) lying-in charities were invited, to discuss practical remedies for this state of affairs; and the interim report states that there is good reason to hope that before long it will be possible for respectable women to be received into voluntary hospitals instead of Poor-Law institutions, notwithstanding that they may be infected with venereal disease.

### THE EXPANSION OF HOSPITALS: THE NEW PHASE.

The word "extension" is becoming less and less applicable to the new undertakings which hospital managers have now to plan and provide. An era of expansion has begun with their reception of wounded soldiers, and we may expect not merely reduced waiting-lists but a higher percentage of beds per thousand of the population once peace comes to give a free head to the movement which the needs of the war initiated. The Royal Victoria Infirmary, Newcastle, to take a significant instance, is planning a new department for cases of venereal disease, a skin department, a department of massage, and a general reduction of the overcrowding from which it cannot escape at present. Translated into figures, this may involve an outlay of £60,000; but, critically examined, it is seen to be the start of the turn of the tide, which finds a parallel movement in practically every important centre. The appeal for "future needs" is heard everywhere, and a new phase begins in its evolution of the voluntary system.

### THE INSTITUTIONAL MEATLESS DAY.

Will any forthcoming legislation, or application of existing legislation, in respect of meatless days apply to such institutions as hospitals, where there are always a number of people whose diet, as directed by medical men, includes meat prepared in one form or another? Even if the new restrictions do so apply it is probable that certain patients will continue to eat meat on the specified days if their condition so requires it, on the same principle that brandy may be purchased outside specified hours on the requisition of a medical man. In respect of patients on full diet, and of the staff inmates of an institution generally, joints of meat are from the point of view of convenience by far the most easily handled and cooked form of food for the chief meal, to replace which would require a great deal of consideration. Hospitals may have to take a leaf out of the book of institutions under the Lunacy Authorities, where the problem has already been partly solved. It is our duty to examine a number of the reports of the asylums, in which specimen meals are often described which may serve as a guide to choice should difficulty arise. One dinner consisted of "good helpings of boiled rice and rhubarb tart, with bread and cheese," and such a mid-day meal as pea-soup followed by suet puddings and jam or treacle is not unusual. To the average hospital worker such a diet no doubt sounds curious, but it is satisfying and wholesome, and the instances we have quoted have been approved by the Lunacy Commissioners.

### THE PREMIER AND WELSH AMBULANCE WORK.

The Prime Minister has consented to become president of the St. David's Centre of the St. John Ambulance Association in succession to Lord Dynevor. The Association has lately been occupied with the problem of providing the 500 additional beds in the Western Command which have been asked for by the Deputy Director of the Medical Service. It is noteworthy, too, that the State of Newfoundland has expressed a wish for the endowment of a ward in Porthcawl Hospital, in which all Newfoundlanders will be placed. The ward will, of course, be known as the Newfoundland Ward. These incidents by no means exhaust the activities of ambulance work in Wales, and the expansion of activity indicated by them is satisfactorily recognised by the Premier's consent to act as president of the St. David's Centre.

### ARCHITECTURE AND PUBLIC HEALTH.

Mr. Paul Waterhouse, whose experience as an architect both in lay and institutional buildings has given him a maturer judgment than that of most of his professional colleagues, has done a public service in his course of lectures on "Architecture in relation to Health and Welfare." While he is as much alive as Ruskin himself to the fact that the appeal of Nature to the imagination in the country must be complemented by the appeal of architecture in the town, he begins at the hard practical difficulties of road-making, and realises as a first principle (so often neglected in town-planning schemes) that to provide for future developments is as strict a duty to-day as to plan a city was in the Middle Ages. Since change is certain, it is our own duty to prepare for it, so that order and economy, the roots of beauty, may be secured in advance. Our cities to-day are poisonous congeries of slums, because their extension has been left to chance. London, he pointed out, needs for health and convenience a girdle road between the rim of its centre and its suburbs; and one of Mr. Waterhouse's most comprehensive suggestions was that this girdle should be not a thoroughfare only, but a belt of that woodland which even the jerry-builder has in some degree spared at many points on the circuit which runs between, say, Hampstead and Richmond. Good design in architecture is really a branch of public health, and Mr. Waterhouse's grasp of this essential truth has given insight into a suggestive series of lectures which the late Sir Edwin Chadwick would have appreciated to the full.

### THE NEW HAWKMOOR SANATORIUM.

The Devonshire County Council's scheme for the treatment of tuberculosis has reached the institutional stage with the opening of the Hawkmoor Sanatorium. The new sanatorium is largely due to the Medical Officer of Health, Dr. George Atkins, working in conjunction with the ex-Chairman of the Public Health Committee, Dr. Lewis Mackenzie, and Sir Robert Newman. Probably every visitor to Devon who has not confined himself to the sea coast knows the neighbourhood of Bovey Tracey, on the Moretonhampstead Road. It is here that the sanatorium is placed, in the heart of the Hawkmoor Estate, 345 acres in all, which was purchased in 1913 for £9,250. Of this acreage the sanatorium enjoys 134, on which forty chalets have been occupied by patients during the building of the sanatorium itself. This accommodates eighty beds, apart from the children's pavilion, known as the sanatorium school, where fifty beds have been provided. The cost per bed averages about £200. Hawkmoor claims to be the first sanatorium actually built by a County Council to comply with the provisions of the Insurance Act. Dr. Penn Milton, who was appointed medical superintendent, is absent on war service, and his place is being taken by Dr. Tucker Wise. The matron is Miss Powell, who was sister at the County Hospital, while Miss Panter, from Pinhoe Sanatorium, is her assistant. The architect is Mr. J. F. Bowden, of Exeter.

### "TUBS FOR TOMMIES."

Allusion has already been made in these columns to the fund raised by the Emergency Voluntary Aid Committee of the Empress Club to provide hot bathing equipment for the use of our much-tried troops in France. According to a statement recently issued, over 2,100 of these portable baths, complete with heating apparatus, towels, soap, scrubbers, and disinfectors (for verminous clothing), have been supplied to the Army in full working order. So greatly are they appreciated that an appeal is issued for funds sufficient to supply 5,000 more. It is pointed out that the sum of £10 provides a unit of five baths, with stove, boiler, and all equipment. Each unit of five baths will suffice to give 100 men per day a much-needed hot bath, a comfort which at the Front is far more necessary to health and fitness than it ever is at home. In aid of this fund, whose headquarters are at 48 Old Bond Street, a sixpenny puzzle is being sold, in connection with which a competition for a money prize is advertised: this prize is the only feature about the "Tubs for Tommies" fund which does not command our complete admiration and approval.

### THE PROBLEM OF PREMISES.

THE South London Hospital for Women is not the only recent institution of its class which is facing the problem of extension. At Nottingham the Women's Hospital is in need of better premises to bring the institution into line with the modern hospital standard and to increase the number of occupied beds beyond the present humble figure. There is a stage in the growth of most institutions when the aim has been put forward rather than achieved, and we can recall one in the Metropolis the premises and front of which are a ridiculous expression of the work carried on within its walls. Under such circumstances a new building is a good investment, since an institution which is badly housed does not attract support in full measure. But a great effort is required on the part of those who share its aspirations if the financial corner is to be turned, and the work placed upon a proper basis.

### THE CAVELL STATUE.

AT the point where St. Martin's Lane and Charing Cross Road meet, an island site has been found for the erection of a monument of Nurse Cavell, of which Sir George Frampton, R.A., is the sculptor. The clay figure and the scale model were recently inspected and approved. The figure, designed in uniform, stands against a monolith, and in relation to its background, as in respect of its size, affords the effect of a group, the monument being surmounted by a figure of Humanity holding a child. The ground plan of the whole design, as every nurse will note, is that of the Geneva Cross. The site is a fine one, commanding the famous church, two thoroughfares, and the square which London has dedicated to its naval and military heroes. The cost of the scheme is being met by subscriptions to the *Daily Telegraph* Fund.

### VEGETABLE CULTIVATION FOR HOSPITALS.

No statement has, we believe, so far been made as to whether applications will be favourably received for grants of waste land to institutions for the growing of annual crops of vegetables, etc. under the new allotment scheme, but the opportunity seems a good one. We have often in these columns emphasised the value of the hospital garden and advocated the cultivation of land around hospitals from the utilitarian point of view. In one V.A.D. hospital near London a most valuable crop of vegetables has been and is being now obtained, thanks very largely to the work of the convalescent patients upon a piece of land adjoining the institution. Let other institutions apply for grants of waste land and follow this example.

### HOSPITAL SERVANT PROBLEM.

ELECTRIC cooking and heating are to be tried to solve the servant problem. At the last meeting of the Hospital Committee of the Swansea Corporation the Medical Officer of Health reported that difficulty was being experienced in getting servants for the hospital, and he recommended the installation of electrical apparatus for cooking and heating. This would be of great assistance in times of stress and shortage of staff. It was agreed that the electrical engineer should carry out the work, which, it was mentioned, would cost about £20.

### "THE FIFTH."

THE first number of the monthly gazette to be known as *The Fifth*, which is issued by the patients and staff of the 5th (City of London) General Hospital, otherwise St. Thomas's, has now been published, with a cover-design by Stuart Lawson. The price is 3d. We await a copy for review.

### THIS WEEK'S DRUG MARKET.

BUSINESS has been practically at a standstill since the last report appeared, but we may expect to see a revival early in the New Year. The year that is now drawing to a close has witnessed fewer of those remarkable fluctuations in value which were a feature of the market last year. Prices have to some extent passed through a period of adjustment. A number of drugs are cheaper now than they were a year ago; among them are salicylic acid, salicylate of soda, acetylsalicylic acid, and various other synthetic drugs; bromides and ipecacuanha are other notable instances of drugs the value of which has dropped very substantially. On the other hand, there is at least an equal number of drugs that are much dearer than they were a year ago, as, for instance, phenacetin, barbitone, resorcin, chamomiles, and opium. So far as one can foresee, the best advice to offer to drug-buyers —at any rate for the immediate future—is to act with caution and to purchase only sufficient for short-period requirements.

### TO OUR READERS.

**CONTRIBUTIONS are specially invited from any of our readers to these columns. They should deal with topical subjects and news. They must be authenticated for the information of the Editor only. The minimum payment if published is 5s. There is no hard-and-fast rule as to space, but notes of about twenty lines in length are preferred.**

